# Cytosolic Triosephosphate Isomerase from *Arabidopsis thaliana* Is Reversibly Modified by Glutathione on Cysteines 127 and 218

**DOI:** 10.3389/fpls.2016.01942

**Published:** 2016-12-22

**Authors:** Sébastien Dumont, Natalia V. Bykova, Guillaume Pelletier, Sonia Dorion, Jean Rivoal

**Affiliations:** ^1^Institut de Recherche en Biologie Végétale, Département de sciences biologiques, Université de MontréalMontréal, QC, Canada; ^2^Morden Research and Development Centre, Agriculture and Agri-Food CanadaMorden, MB, Canada

**Keywords:** cytosolic triosephosphate isomerase, *Arabidopsis thaliana*, *S*-glutathionylation, deglutathionylation, glutaredoxin, redox post-translational modification, cysteine

## Abstract

In plant cells, an increase in cellular oxidants can have multiple effects, including the promotion of mixed disulfide bonds between glutathione and some proteins (*S*-glutathionylation). The present study focuses on the cytosolic isoform of the glycolytic enzyme triosephosphate isomerase (cTPI) from *Arabidopsis thaliana* and its reversible modification by glutathione. We used purified recombinant cTPI to demonstrate the enzyme sensitivity to inhibition by *N*-ethylmaleimide, hydrogen peroxide and diamide. Treatment of cTPI with diamide in the presence of reduced glutathione (GSH) led to a virtually complete inhibition of its enzymatic activity by *S*-glutathionylation. Recombinant cTPI was also sensitive to the oxidized form of glutathione (GSSG) in the micromolar range. Activity of cTPI was restored after reversion of *S*-glutathionylation by two purified recombinant *A. thaliana* cytosolic glutaredoxins (GRXs). GRXs-mediated deglutathionylation of cTPI was dependent on a GSH-regenerating system. Analysis of cTPI by mass spectrometry after *S*-glutathionylation by GSSG revealed that two Cys residues (Cys127 and Cys218) were modified by glutathione. The role of these two residues was assessed using site-directed mutagenesis. Mutation of Cys127 and Cys218 to Ser separately or together caused different levels of decrease in enzyme activity, loss of stability, as well as alteration of intrinsic fluorescence, underlining the importance of these Cys residues in protein conformation. Comparison of wild-type and mutant proteins modified with biotinyl glutathione ethyl ester (BioGEE) showed partial binding with single mutants and total loss of binding with the double mutant, demonstrating that both Cys residues were significantly *S*-glutathionylated. cTPI modification with BioGEE was reversed using DTT. Our study provides the first identification of the amino acid residues involved in cTPI *S*-glutathionylation and supports the hypothesis that this reversible modification could be part of an oxidative stress response pathway.

## Introduction

In plant cells, ROS such as superoxide anion ($O_{2}^{•-}$), hydrogen peroxide (H_2_O_2_) and hydroxyl radical (OH^•^) are normal by-products of many cellular processes like photosynthesis (Karpinski et al., 2003; Apel and Hirt, 2004) and aerobic metabolism (Miwa and Brand, 2003). In addition, many biotic and abiotic stresses can lead to abnormal accumulation of ROS (Inzé and Montagu, 1995; Mittler, 2002) which can potentially damage cell structures and impair metabolic activities through oxidation of biomolecules such as nucleic acids, fatty acids and proteins (Gill and Tuteja, 2010). Due to their nature, ROS also play important roles in plant redox signaling under basal conditions and during stress response by triggering defense mechanisms against oxidative stress (Foyer and Noctor, 2009).

Cysteine thiol groups are sensitive to oxidation by ROS due to their electron-rich sulfur center, making Cys residues potential targets of various redox PTMs (Davies, 2005). In cells, the reactivity of Cys thiol groups increases through stabilization of the thiolate anion in the Cys microenvironment. This can occur for example because of the proximity of basic amino acids. Thiolate anions are much stronger nucleophiles than protonated thiol groups. They are more prone to undergo ROS-mediated oxidation such as the formation of sulfenic acid thiol and other redox PTMs such as *S*-nitrosylation and *S*-glutathionylation (Bykova and Rampitsch, 2013). Protein *S*-glutathionylation results from the reaction of sulfenic acid thiol with GSH or by thiol disulfide exchange between a reduced thiol group and GSSG leading to the formation of a mixed disulfide bond between glutathione and the targeted protein (Dalle-Donne et al., 2009; Zaffagnini et al., 2012d). Protein *S*-glutathionylation was first described as a mechanism to protect Cys residues from irreversible oxidation (Klatt and Lamas, 2000; Bedhomme et al., 2012). Recently, *S*-glutathionylation has also emerged as part of different redox signaling pathways in many organisms including plants (Gallogly and Mieyal, 2007; Pastore and Piemonte, 2012). Indeed, PTM of Cys by glutathione can alter the three-dimensional structure of targeted proteins and modify enzymes activity, thus affecting specific biochemical pathways (Zaffagnini et al., 2012d). *S*-glutathionylation occurs spontaneously *in vitro* but was also reported to be catalyzed by glutathione *S*-transferases (GSTs) of the π class (GSTπ) in animals (Townsend et al., 2009) and by a GRX in *Arabidopsis thaliana* (Bender et al., 2015). Despite *in vitro* evidence for catalysis of protein *S*-glutathionylation, the main mechanism leading to this modification *in vivo* remains unclear. Nevertheless, treatments with GSSG, GSH + H_2_O_2_, and GSH + diamide have been used to study protein *S*-glutathionylation *in vitro* (Barrett et al., 1999; Ito et al., 2003; Holtgrefe et al., 2008; van der Linde et al., 2011; Bedhomme et al., 2012; Butturini et al., 2014; Zaffagnini et al., 2014). *S*-glutathionylation is reversible and protein deglutathionylation is mainly catalyzed by GRXs. GRXs are small thiol-disulfide oxidoreductase belonging to the thioredoxin superfamily. Class I (CPYC-like active site) and class II (CGFS active site) GRXs are present in almost all living organism (Rouhier et al., 2008; Zaffagnini et al., 2012b). A third class, CC-type GRXs, is only present in higher plants and their biochemical properties are still largely unknown. Class I GRXs are the most recognized for their protein deglutathionylation functions in plants (Zaffagnini et al., 2008; Gao et al., 2010; Bedhomme et al., 2012; Zaffagnini et al., 2012c). *A. thaliana* possesses two cytosolic class I GRXs: GRXC1 and GRXC2 and both can reduce peroxiredoxin IIB (Riondet et al., 2012). The poplar (*Populus trichocarpa*) ortholog of *A. thaliana* GRXC1 has been shown to perform *in vitro* deglutathionylation of cytosolic glyceraldehyde 3-phosphate dehydrogenase (GAPC1) (Bedhomme et al., 2012). Hence, GRXC1 and GRXC2 might play major roles in deglutathionylation of plant cytosolic enzymes *in vivo.*

In photosynthetic organisms, more than 200 *S*-glutathionylation targets have been identified using different types of approaches (Dixon et al., 2005; Michelet et al., 2008; Zaffagnini et al., 2012a). Proteins identified in these surveys are part of many metabolic pathways including glycolysis and the Calvin–Benson cycle. Triosephosphate isomerase (TPI; EC 5.3.1.1) is a housekeeping enzyme that plays essential role in glycolysis by catalyzing the interconversion between GAP and DHAP. Photosynthetic organisms have a chloroplastic isoform (pTPI) that also participates in photosynthetic carbon metabolism. The *S*-glutathionylation of *A. thaliana* cTPI was shown to inhibit enzyme activity (Ito et al., 2003). More recently, pTPI from *Chlamydomonas reinhardtii* was also shown to be *S*-glutathionylated *in vitro*, with a negative impact on its activity (Zaffagnini et al., 2014). It is important to note that these results were obtained with relatively high concentrations (2–2.5 mM) of GSSG. Other evidence for redox modification of the cytosolic isoform is the interaction between cTPI and TRXs in *A*. *thaliana* roots and leaves (Marchand et al., 2004, 2010). Analysis of the TPI-TRX complex from *Plasmodium falciparum* suggested that TRX could perform deglutathionylation on *Pf*TPI Cys217 (Shahul and Sarma, 2011).

In human, TPI deficiency (OMIM: 615512) is a rare genetic disorder leading to premature death and is the object of intensive research (Orosz et al., 2009). Interestingly, low TPI activity has been associated with higher resistance to the thiol-oxidizing reagent diamide in yeast (Ralser et al., 2006) and *Caenorhabditis elegans* (Ralser et al., 2007). It has been demonstrated that cells with reduced TPI activity have a higher NADPH/NADP^+^ ratio due to an increase in carbon flux through the PPP, thus providing more reducing power within the cytosol (Ralser et al., 2007). In plants, transgenic potato roots with reduced cTPI activity also showed a greater carbon flux through the PPP compared to controls (Dorion et al., 2012). Metabolic modeling simulations also suggested a rerouting of carbon flux through PPP in plant cells with low cTPI activity (Valancin et al., 2013). These studies strongly suggest that cTPI as well as other glycolytic enzymes can partake in a cellular switch to influence the cytosolic redox status by redirecting the carbon flux through the PPP.

In the present study, we show that activity of *A. thaliana* recombinant cTPI is sensitive to inhibition by oxidation and *S*-glutathionylation. We also demonstrate that *S*-glutathionylation of cTPI is reversible by *A. thaliana* GRXC1 and GRXC2. We use nanoLC-MS/MS analysis to identify two Cys residues targeted by modification with glutathione in cTPI. Analysis of purified site directed mutant proteins for these Cys residues suggests differences in enzyme activity, structure and stability. This indicates that *S*-glutathionylation targets residues that are important for cTPI structure and function.

## Materials and Methods

### Chemicals

BioGEE was purchased from Molecular Probes (Eugene, OR, USA) or synthesized from 25 mM EZ-Link-Sulfo-NHS-Biotin (Thermo Fisher Scientific, Nepean, ON, Canada) and 25 mM glutathione ethyl ester (Sigma Chemical Co., St Louis, MO, USA) in 50 mM phosphate buffered saline at pH 8. After 1 h incubation, NH_4_HCO_3_ was added to a final concentration of 138 mM to quench the remaining EZ-Link-Sulfo-NHS-Biotin. The concentration of the newly synthesized BioGEE was determined spectrophotometrically with 5,5′-dithiobis-(2-nitrobenzoic acid) using GSH as standard. Except when indicated otherwise, buffers, chemicals, and reagents were of analytical grade from Sigma Chemical Co. or Thermo Fisher Scientific.

### Plasmid Constructions and Site Directed Mutagenesis

Standard techniques were used for recombinant DNA manipulations (Sambrook et al., 1989). *A. thaliana* sequence for cTPI (At3g55440) and GRXC1 (At5g63030) in pUNI51 vector, and GRXC2 (At5g40370) in PENTR/SD-DTOPO vector, were obtained from ABRC (respective stock numbers U12445, U16036, and U21122) and amplified by PCR using the following primers: cTPI forward 5′ATGGCCAGAAAGTTCTTCGTC3′ and reverse 5′TGTTAGCAGCCGGATCTTCTA3′; GRXC1 forward 5′ATGGGTTCAATGTTCAGTGGA3′ and reverse 5′ATTATAGCGGCCGCTACAAGAAAGCTGGGTCGG3′; GRXC2 forward, 5′ATGGCGATGCAGAAAGCTAAG3′ and reverse 5′TGGCTGGCAACTAGAAGGCAC3′.

The resulting amplicons were purified using a QIAquick gel extraction kit (Qiagen, Chatsworth, CA, USA) and digested by *NotI.* All digested inserts were cloned into the expression vector pProEx HTb (Invitrogen Canada Inc., Burlington, ON, Canada) previously digested with *EheI* and *NotI*. Restriction digestions were used to confirm the presence of the insert. The ligated plasmids were used to transform competent *Escherichia coli* cells (HB101 strain for cTPI and GRXC2 or Rosetta^TM^ strain for GRXC1). cTPI Cys residues detected to be involved in mixed disulfide modification were modified to Ser to form three mutants (C127S, C218S, and C127/218S). Mutagenesis was performed by NorClone (London, ON, Canada) on pProEx HTb containing the cTPI insert. All the constructions used in this study were verified by full sequencing. WT designates the original cTPI protein.

### Production and Purification of Recombinant Enzymes

Recombinant proteins were expressed in *E. coli* as previously described (Dorion et al., 2005) except that cells were grown overnight at room temperature for cTPI or 4 h at 37°C after the addition of 0.6 mM isopropyl β-D-thiogalactoside to the medium for GRXC1 and GRXC2. The purification of recombinant enzymes was performed under native conditions according to the manufacturer’s instructions (Invitrogen Canada Inc, Burlington, ON, Canada). Purified fractions were pooled and dialyzed against a buffer containing 25 mM Tris-Cl pH 7.5, 1 mM MgCl_2_ and 10 mM DTT. To remove DTT, samples were dialyzed twice against the same buffer containing no DTT. The purified and dialyzed enzymes were stored at -20°C in 50% (v/v) glycerol and were stable for over 3 months without any significant loss of activity. Different aliquots of the stored enzymes were used as material for independent experiments.

### cTPI Activity Assays, Kinetic Analysis, and Protein Concentration

cTPI activity was measured by following the isomerization of GAP to DHAP at 30°C using a coupled enzyme assay on a VERSAmax microplate reader (Molecular Devices, San Diego, CA, USA) (Dorion et al., 2005). GAP used as a substrate was prepared from DL-GAP diethyl acetal barium salt (G5376, Sigma Chemical Co., St Louis, MO, USA) according to the procedure provided by the supplier. cTPI assays mixture contained 100 mM Tris-Cl pH 7.8, 1 mM GAP, 0.5 mM EDTA, 2.5 U/mL α-glycerophosphate dehydrogenase and 0.2 mM NADH. For stability assays, WT and mutant proteins were incubated at room temperature (21–23°C) with 100 mM Tris-Cl pH 7.8, 0.005% (w/v) BSA with or without 5 mM DTT and activity was measured after different incubation times. After 145 min, the samples without DTT were treated with 10 mM DTT for 30 min and TPI activity was measured again. To determine *K*_m_ and *k*_cat_ values, activity assays were performed with varying concentrations of GAP (0–4 mM) in the presence of 5 mM DTT. *K*_m_ for GAP was determined using a non-linear regression analysis software (SigmaPlot 8.0, SPSS Inc., Chicago, IL, USA). Protein concentration was measured by the method of Bradford using BSA as a standard (Bradford, 1976).

### Electrophoresis, Immunoblotting, Immunodetection, and Activity Staining

SDS-PAGE analysis was performed according to Laemmli (1970) on 15% (w/v) polyacrylamide gels. For immunoblotting, gels were transferred onto a nitrocellulose membrane for 60 min at 70 V. Immunodetection of cTPI was carried out with rabbit anti-cTPI polyclonal immune serum (1/500 dilution) (Dorion et al., 2005). The detection was done using a goat anti-rabbit IgG conjugated to alkaline phosphatase secondary antibody (1/10,000 dilution) (Promega, Nepean, ON, Canada). Native PAGE was performed on 10% (w/v) acrylamide gels according to Laemmli (1970) except that gels and electrode buffers were exempt of SDS. cTPI activity staining was performed essentially according to a previously described method (Dorion et al., 2005). Control gels incubated without GAP did not show any activity band. Detection of BioGEE was performed using streptavidin conjugated with alkaline phosphatase (Molecular Probes, Eugene, OR, USA) using a 1/40,000 dilution for reducing conditions and 1/20,000 for native conditions.

### GRXs GSH-Disulfide Transhydrogenase Activity Assay

The protocol to assay GRX activity using HED as substrate was adapted from Zaffagnini et al. (2008). Briefly, various amounts of GRXs (0.05 to 0.4 μg/ml final concentrations) were added to a reaction mixture containing 100 mM Tris-Cl pH 8.0, 2 mM EDTA, 0.01% (w/v) BSA, 1 mM GSH, 0.2 mM NADPH, 6 μg/ml yeast glutathione reductase and 0.7 mM HED in a final volume of 200 μl. Reactions were carried out in 96-well microplates at 30°C and NADPH disappearance over time was measured at 340 nm on a microplate reader. Background activity measured in the absence of GRX was subtracted from activity obtained in the presence of GRX. GRX activity was expressed as μmoles of NADPH oxidized/min and was proportional to the quantity of GRX added.

### Sensitivity of cTPI to H_2_O_2_, Diamide, NEM, and GSSG

Purified recombinant cTPI (0.2 μg/ml) was incubated at room temperature with 100 mM Tris-Cl pH 7.8, 0.005% (w/v) BSA with or without addition of 1 mM GSH and then treated with 0.5 mM H_2_O_2_, 1 mM diamide or 0.5 mM NEM. TPI activity was determined at different incubation times. After 120 min, DTT was added to all the samples at a final concentration of 10 mM and activity was measured 30 min later. To test the immediate effect of diamide on cTPI, the purified enzyme was first incubated with 100 mM Tris-Cl pH 7.8, 0.005% (w/v) BSA with or without addition of 1 mM GSH or 1 mM GSSG. The activity was determined before and within 5 min of addition of diamide at a final concentration of 1 mM. DTT was then immediately added to a final concentration of 10 mM for 90 min. For inhibition of cTPI by GSSG, purified cTPI (0.2 μg/ml) was incubated in 100 mM Tris-Cl pH 7.8, 0.005% (w/v) BSA with different concentrations of GSSG (0–2.5 mM). TPI activity was assayed after various times of incubation.

### Reactivation of cTPI Activity by GRX-Mediated Deglutathionylation

For BioGEE detection on nitrocellulose membranes, recombinant cTPI (75 μg/ml) was incubated at room temperature with 100 mM Tris-Cl pH 7.8, 0.5 mM BioGEE and 0.5 mM diamide to induce spontaneous *S*-glutathionylation. After 5 min incubation, the cTPI sample was desalted using a PD10 column to remove excess of diamide and BioGEE. The desalted cTPI sample was then divided into different incubation mixtures containing 100 mM Tris-HCl pH 7.8, 0.01% (w/v) BSA (CTL), or with the addition of a GRS (2 mM EDTA, 1 mM GSH, 0.2 mM NADPH, 0.6 μg/ml glutathione reductase) or with 10 μM of recombinant GRXs (GRXC1 or GRXC2 samples), or with the addition of both GRS and GRXs (GRS + GRXC1/C2 samples) or with 10 mM DTT. Samples were incubated at room temperature for 30 min. An aliquot of the different treatments was used for Native-PAGE analysis and another aliquot was denatured for 5 min at 94°C for SDS-PAGE analysis. Electrophoresis and immunoblotting were performed as described above. For cTPI reactivation assays, recombinant cTPI (5.3 μg/ml) was incubated with 0.005% (w/v) BSA, 100 mM Tris-HCl pH 7.8, 1 mM GSH and 1 mM diamide for 5 min and desalted as described above. A non-glutathionylated control was also prepared identically but without addition of diamide. Aliquots of the desalted cTPI sample were used in the same treatments as described for the BioGEE detection except that 0.2 μM of GRXs was used. An aliquot of the different samples was taken at different time points to measure cTPI activity.

### Fluorescence Emission Spectra

Fluorescence was determined using a SpectraMax M5 Multi-Mode Microplate Reader (Molecular Devices, San Diego, CA, USA). Recombinant enzymes were diluted (0.1 mg/ml) in 100 mM Tris-Cl pH 7.8 and fluorescence emission spectra were recorded using an excitation wavelength of 274 nm.

### Effect of GSSG on WT and Mutant cTPI Electrophoretic Mobility

The recombinant proteins (5 μg/ml) were incubated for 120 min with 100 mM Tris-Cl pH 7.8, 1 mM GSSG or with 100 mM Tris-Cl pH 7.8, 15 mM DTT. Samples were then denatured for 5 min at 94°C. Immunoblot analyses against cTPI were performed as described above.

### BioGEE Labeling of Recombinant Proteins

Recombinant proteins (5 μg/ml for SDS-PAGE and 20 μg/ml for Native-PAGE) were incubated for 120 min with 100 mM Tris-Cl pH 7.8, 0.5 mM BioGEE, 0.1 mM H_2_O_2_ for native gels and with the addition of 0.005% (w/v) BSA to the incubation mix for denaturing gels. As a control, 50 mM DTT was added to the loading buffer. Samples for SDS-PAGE were denatured for 5 min at 94°C and analyzed by immunoblotting.

### Trypsin Digestion and NanoLC-MS/MS Analysis

For determination of modified Cys residues, cTPI (100 pmol) was incubated with 2.5 mM GSSG or left untreated as a control and dialyzed at room temperature overnight against 100 mM NH_4_HCO_3_. In-solution protein digestion was carried out overnight at 37°C in 100 mM NH_4_HCO_3_ buffer supplemented with 10% (v/v) acetonitrile, 2.5 mM CaCl_2_ and trypsin (modified, sequencing grade, Promega, Madison, WI, USA) in a trypsin-to-protein ratio 1:50, followed by drying down in a speed vac. In control experiments GSSG-treated and untreated cTPI proteins were also reduced with 30 mM DTT at 56°C for 45 min and alkylated at room temperature in the dark with 70 mM iodoacetamide prior to dialysis and trypsin digestion. All samples were re-dissolved in loading buffer containing 1% (v/v) formic acid and 0.5% (v/v) acetic acid. Automated nano-flow LC-MS/MS analysis of peptide digests was performed using a linear ion trap Finnigan LTQ XL (Thermo Finnigan, San Jose, CA, USA) mass spectrometer connected on-line with nano-HPLC (Dionex UltiMate^TM^ 3000). The chromatographic separation was accomplished with a 10 cm reversed-phase nano-column (75 μm OD, 360 μm OD; packed in-house with Vydac C18, 5 μm bead and 300 Å pore size resin). The flow rate was set at 250 nl/min and peptide elution was performed using a linear gradient of 4–40% (v/v) ACN for 44 min, followed by a gradient of 40–80% (v/v) ACN for 3 min, and 80% (v/v) acetonitrile for 2 min in 1% (v/v) formic acid, 0.5% (v/v) acetic acid. The mass spectrometer was operated in the positive ion mode with the source temperature set at 200°C. It was tuned in nano-spray mode using 10 μM (Glu)-Fibrinopeptide B singly charged ion at m/z 1552.67. Data-dependent analysis was employed with one single MS full survey scan set at m/z range 450–2000 followed by CID MS/MS product ion scans of the five most abundant ions in each cycle with 20 s dynamic exclusion. The results were reproduced, and two independent runs were performed for each sample with 10 and 36 pmol total protein loaded per LC-MS/MS analysis.

### Protein Identification and Assignment of Modification Sites

Peptide CID fragmentation spectra were searched using Mascot v. 2.4 search engine (Matrix Science, London, UK) against the NCBInr protein database (*A. thaliana*) first using a general ID search followed by the Error Tolerant search to allow for protein modification screening. The raw data created by XCalibur (Thermo Scientific) version 2.2 on LTQ-XL instrument were converted into Mascot Generic Format with Mascot Distiller 2.4 (Matrix Science, London, UK) software and used for peptide/protein identification and modification screening. The Mascot MS/MS Ion Search parameters were as follows: (1) tryptic digest with maximum one missed cleavage; (2) monoisotopic peptide masses were used; (3) the peptide mass tolerance was kept at 2 Da; and the fragment ions mass tolerance was set at 0.8 Da; (4) variable modifications Glutathione (C), Carbamidomethyl (C), Pyro-carbamidomethyl (N-term C), Oxidation (M), and Deamidation (NQ) were used; (5) peptide charge state +1, +2, and +3 for LC-MS/MS spectra. A single peptide probability of identification MOWSE score greater than 42 for cTPI experiments indicated identity or extensive homology (*p* < 0.05). Spectra with modifications were verified manually using the GPMAW 9.2 (Lighthouse Data, Odense, Denmark) software.

### Replication and Statistical Analysis

Data were analyzed using the unpaired Student *t*-test tool of SigmaPlot 8.0, with *P* < 0.05 considered as a significant difference. The number of independent experiments (*n*) is indicated in each table or figure. Otherwise, figures show representative data of at least three experiments. Each independent experiment was the mean of at least three technical replicates. Several protein purifications were used during this work with low variations between purification batches (<10% variation in specific activity).

## Results

### Purification of Recombinant His-Tagged cTPI from *A. thaliana*

**FIGURE 1:**
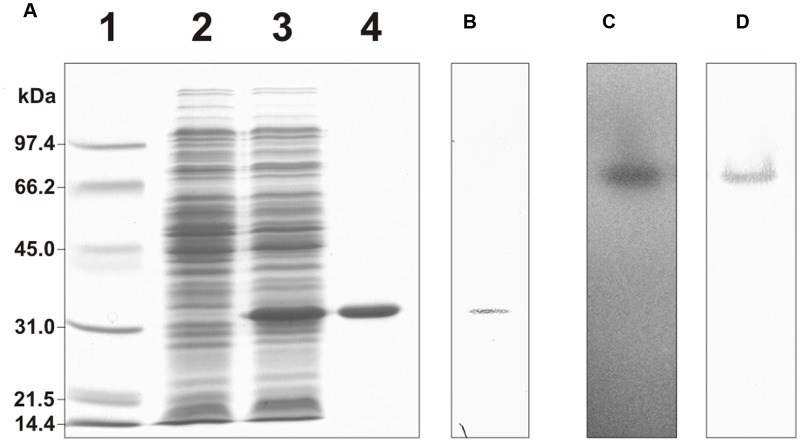
**Purification of His-tagged recombinant cTPI. (A)** SDS-PAGE analysis of the purification of recombinant cTPI. Lane 1, molecular weight standards; lane 2, *E. coli* protein extract without induction; lane 3, *E. coli* protein extract after isopropyl β-D-thiogalactoside induction; lane 4, affinity-purified recombinant cTPI. **(B)** Western blot analysis of the purified recombinant cTPI (25 ng) using an isoform-specific antibody against cTPI. **(C)** Native gel electrophoresis of the purified recombinant cTPI (50 ng) followed by TPI activity staining. **(D)** Western blot analysis of **(C)** using anti-cTPI immune serum.

His-tagged cTPI was expressed in *E. coli* and affinity-purified. In the presence of DTT, the recombinant protein migrated as a single band after SDS-PAGE (**Figure 1A**) and was recognized by a rabbit anti-cTPI polyclonal immune serum on a Western blot (**Figure 1B**). Recombinant cTPI was also visualized after native gel electrophoresis and activity staining (**Figure 1C**). The native gel showed a single band of activity which was recognized by the anti-cTPI immune serum (**Figure 1D**).

### Inhibition of cTPI Activity by Oxidants and *S*-Glutathionylation

**FIGURE 2:**
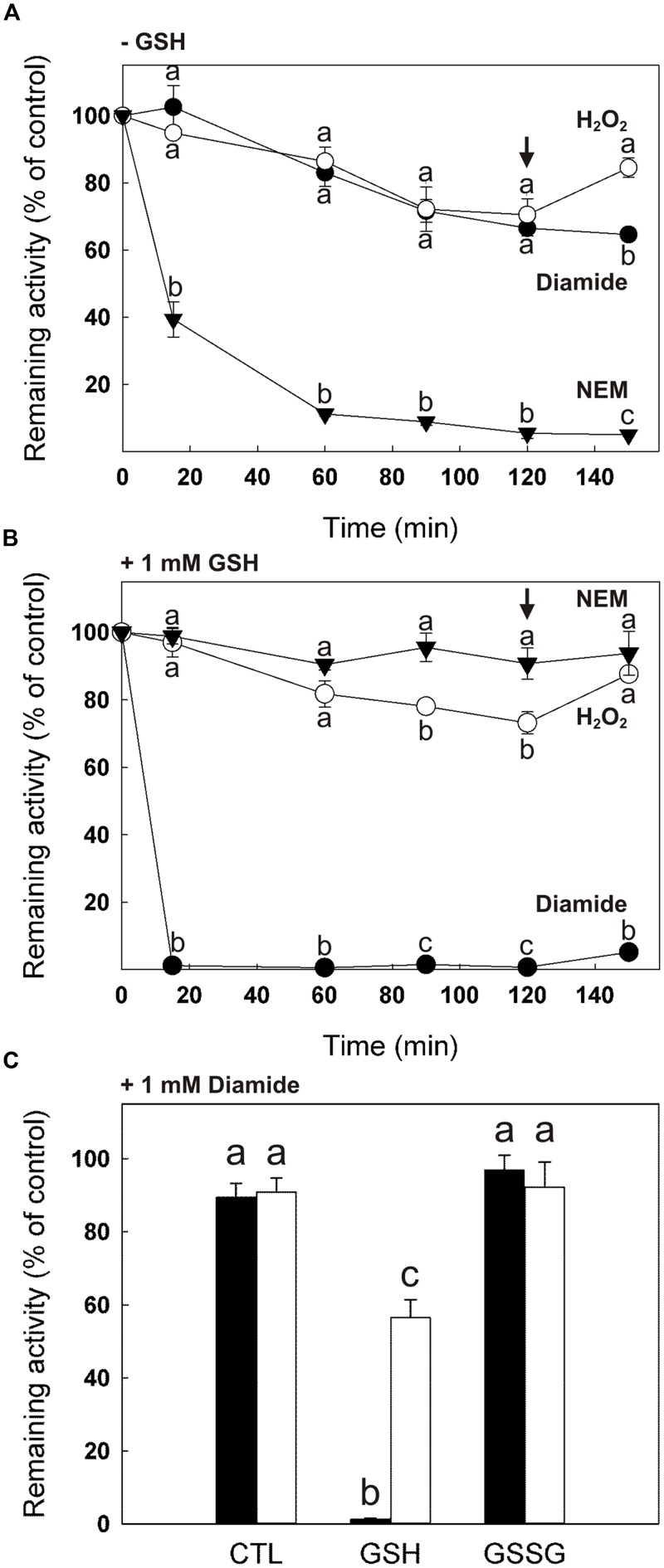
**Effect of GSH and GSSG on the sensitivity of cTPI activity to NEM, H_2_O_2_ and diamide.** Recombinant cTPI was incubated without **(A)** or with **(B)** 1 mM GSH prior to treatment with NEM (*black triangles*), H_2_O_2_ (*white circles*) or diamide (*black circles*). Activity (mean ± SE, *n* = 3) was measured at different time points during the treatment and DTT (10 mM) was added to all samples after 120 min (*black arrow*). In **(C)**, cTPI was incubated with 1 mM GSH (GSH), 1 mM GSSG (GSSG) or no addition (CTL). Samples were treated with 1 mM diamide and cTPI activity (mean ± SE, *n* = 3) was measured within 5 min (*black bars*). After, DTT was added at a final concentration of 10 mM and cTPI activity was measured again after 90 min of incubation (*white bars*). Data with different letters are significantly different (Student’s *t*-test, *P* < 0.05). Activity is expressed as percentage of the control (untreated) sample remaining activity in order to take into account the loss of cTPI activity upon dilution without reductant.

We found that, upon dilution in the absence of DTT, cTPI activity was unstable with incubation time (Supplemental Figure S1). The effect of H_2_O_2_, diamide and NEM on cTPI activity was therefore expressed as percentage of remaining activity of the control to visualize the loss of activity due to the treatment. **Figures 2A,B** show the evolution of cTPI activity over time in the presence of 0.5 mM H_2_O_2_, 1 mM diamide or 0.5 mM NEM with or without addition of 1 mM GSH. Alkylation of cTPI Cys residues by NEM led to a loss of more than 90% in cTPI activity after 60 min of incubation (**Figure 2A**). As expected, addition of 10 mM DTT after 120 min of incubation with NEM did not restore cTPI activity due to irreversible alkylation of its Cys residues. However, in the presence of 1 mM GSH, the addition of 0.5 mM NEM did not affect significantly cTPI activity (**Figure 2B**). This is probably due to the fact that GSH is subject to alkylation and that its concentration in the assay is two times higher than NEM, thus protecting cTPI from being targeted.

Oxidation of recombinant cTPI treated with 0.5 mM H_2_O_2_ alone or in the presence of 1 mM GSH caused about 30% loss of cTPI activity after 120 min compared to the untreated enzyme (**Figures 2A,B**). Addition of DTT after 120 min of incubation partially restored cTPI activity for both samples. Unlike NEM treated samples, presence of 1 mM GSH did not protect cTPI from inactivation by H_2_O_2_, suggesting that cTPI contains Cys residues more sensitive to oxidation.

**FIGURE 3:**
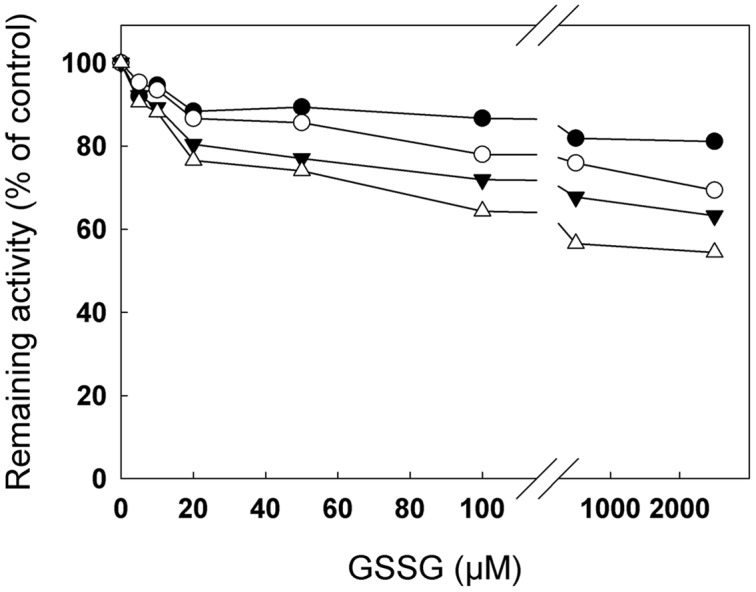
**Inhibition of cTPI activity by GSSG.** Recombinant cTPI was incubated with different GSSG concentrations. TPI activity is represented as a function of GSSG concentration for different incubation times: 75 min (*black circles*); 130 min (*white circles*); 180 min (*black triangles*) and 240 min (*white triangles*). The data are representative of three independent experiments. Activity is expressed as percentage of the control (untreated) sample remaining activity in order to take into account the loss of cTPI activity upon dilution without reductant.

When incubated with the thiol-specific oxidant diamide (1 mM), cTPI activity decreases similarly to the H_2_O_2_ treatment (**Figure 2A**). However, no restoration of cTPI activity was observed when DTT was added to diamide treated samples. Irreversible protein inactivation by diamide has been suggested to be caused by the rapid oxidation of Cys residues into sulfinic and sulfonic acid groups (Bender et al., 2015). cTPI inhibition by diamide was much more pronounced in the presence of 1 mM GSH in the samples (**Figure 2B**). **Figure 2C** shows the effect of treatment of cTPI within 5 min of incubation by diamide alone (CTL), in the presence of 1 mM GSH or 1 mM GSSG. As expected from results in **Figure 2B**, addition of diamide in the presence of GSH caused almost total inhibition of cTPI activity. Incubation for 90 min following the addition of 10 mM DTT partially restored cTPI activity showing that the inhibition is reversible. Interestingly, incubation with diamide + GSSG did not affect cTPI activity within 5 min of incubation. It is to be noted that incubation of GSSG affects negatively cTPI activity, but at longer incubation times (**Figure 3**).

**Figure 3** shows remaining cTPI activity as a function of GSSG concentration for different incubation times. At concentrations as low as 5–10 μM, GSSG produced an observable decrease in recombinant cTPI activity. At much higher concentrations (500–2500 μM) inhibition reached approximately 40% after 240 min of incubation, but the effect of varying GSSG was less substantial on the enzyme activity. Hence, small variations of GSSG in the low concentrations range caused the greatest changes in cTPI activity. The same data plotted as a function of time show that cTPI inactivation by GSSG progresses relatively linearly with time (Supplemental Figure S2). Equal amounts of cTPI (Supplemental Figure S3, right) were subjected to native gel electrophoresis with and without prior to GSSG treatment (Supplemental Figure S3, left). Upon modification of cTPI with GSSG a less intense activity staining was observed. However, it did not change significantly the electrophoretic mobility of the enzyme. These results supported the previous data (**Figure 3**) showing that the GSSG treated cTPI was less active than the control.

### Deglutathionylation of cTPI by Recombinant GRXC1 and GRXC2

**FIGURE 4:**
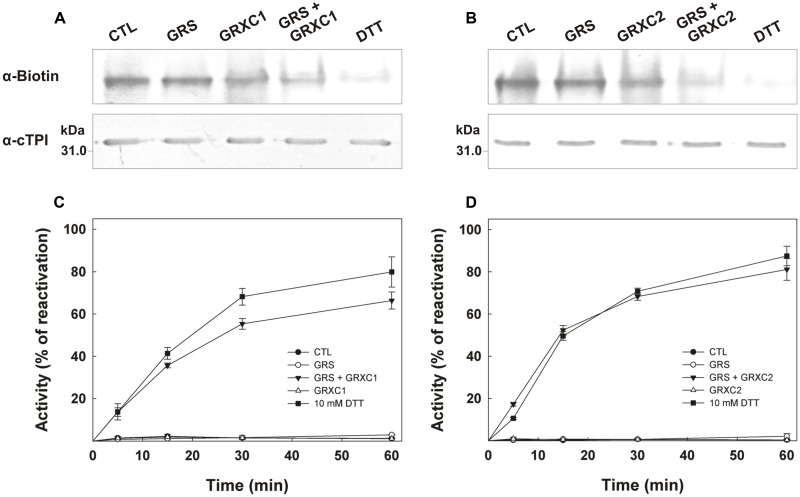
**Deglutathionylation of cTPI mediated by recombinant GRXC1 and GRXC2.** BioGEE detection by streptavidin conjugated with alkaline phosphatase after native PAGE analysis of 350 ng recombinant cTPI **(A,B, upper)**. Recombinant cTPI was *S*-glutathionylated in the presence of BioGEE followed by desalting as described in Section “Materials and Methods.” The enzyme was then treated for 30 min with GSH-regeneration system (GRS), 10 μM **(A)** GRXC1, **(B)** GRXC2 or both GRS + GRXC1 **(A)** or GRXC2 **(B)**. A control sample without treatment (CTL) and another control deglutathionylated in 10 mM DTT (DTT) were also prepared. Aliquots of the samples were denatured and analyzed by Western blot (80 ng proteins) after SDS-PAGE using an anti-cTPI antibody to show equal protein concentrations in samples **(A,B, lower)**. For **(C,D)**, *S*-glutathionylated cTPI (0.2 μg/ml) was incubated with 0.01% BSA (CTL, *black circles*) and GRS (*open circles*) or 10 mM DTT (*black squares*) or 0.2 μM **(C)** GRXC1 **(D)** GRXC2 (*open triangles*) or both GRS + GRXs (*black triangles*). Aliquots of the different treatment were withdrawn at different time points to measure cTPI activity (mean ± SE, *n* = 3). Activity is expressed as percentage of the control (non-glutathionylated) sample.

In order to study the possible deglutathionylation of cTPI by GRXs, His-tagged GRXC1 and GRXC2 from *A. thaliana* were expressed in *E. coli* and affinity-purified (Supplemental Figure S4). The specific activities of recombinant GRXs were measured using the artificial substrate HED as described in material and methods. Calculated specific activities for GRXC1 and GRXC2 were, respectively, 28.8 ± 3.4 μmol min^-1^ mg^-1^ (*k*_cat_ = 8.0 ± 0.9 s^-1^) and 57.7 ± 4.1 μmol min^-1^ mg^-1^ (*k*_cat_ = 14.2 ± 1.0 s^-1^). Both recombinant GRXs were able to catalyze deglutathionylation of cTPI *in vitro* (**Figure 4**). In **Figures 4A,B**, we used BioGEE, a biotinylated glutathione analog and diamide to induce *S*-glutathionylation of recombinant cTPI and observed the impact of different treatments on the enzyme by native gel electrophoresis and BioGEE detection using streptavidin conjugated with alkaline phosphatase on nitrocellulose membranes. The presence of GRS did not affect the intensity of band corresponding to the *S*-glutathionylated cTPI compared to the control. However, addition of GRS + GRXC1 (**Figure 4A**) or GRXC2 (**Figure 4B**) decreased the BioGEE signal compared to the control, GRS alone and GRXs alone, meaning that turnover of GRXs was required to obtain deglutathionylation activity. Incubation with 10 mM DTT was also used as a control to show removal of BioGEE on the native enzyme. Deglutathionylation of cTPI by GRXs was also tested by reactivation of its enzymatic activity after virtually complete inhibition by diamide + GSH treatment (**Figures 4C,D**). After cTPI *S*-glutathionylation and desalting, addition of GRS or 0.2 μM GRXs alone did not rescue cTPI activity in both experiments. Addition of both GRS and 0.2 μM GRXC1 (**Figure 4C**) or 0.2 μM GRXC2 (**Figure 4D**) was able to reverse cTPI inhibition in a time dependent manner. The reactivation rates were comparable to those obtained with the addition of 10 mM DTT. Based on the experimental observations shown in **Figure 4**, GRXC2 was slightly more effective than GRXC1 in catalyzing *in vitro* deglutathionylation of cTPI (which is consistent with specific activities measured by HED assays).

### Identification of *S*-Glutathionylated Residues by Mass Spectrometry Analysis

*S*-Glutathionylation of cTPI from *A. thaliana* was further confirmed by mass spectrometry analysis. Purified *S*-glutathionylated recombinant cTPI was digested with trypsin and analyzed by nanoLC-MS/MS. A triply charged ion at *m/z* value corresponding to the predicted *S*-glutathionylated cTPI tryptic peptide ^124^VIACVGETLEER^135^ (at *m/z* of 542.17^3+^) was detected in the recombinant cTPI sample previously treated with GSSG (Supplemental Figure S5). This peptide ion was not found in control samples or *S*-glutathionylated samples that were previously reduced with DTT and alkylated with iodoacetamide (data not shown). This peptide was also found in two other oxidized forms with Cys-SO_2_H sulfinic acid (at *m/z* of 676.23^2+^) and Cys-SO_3_H sulfonic acid (at *m/z* of 683.99^2+^) indicating high reactivity of Cys127 site possibly caused by the local environment (Supplemental Figure S5). Similarly, a triply charged peptide ion corresponding to the sequence ^207^IIYGGSVNGGNCK^219^ with calculated *m/z* value of the *S*-glutathionylated form of 529.92^3+^ was only found in GSSG-treated samples. Since each of these peptides contains only one Cys residue, the data indicate that Cys127 and Cys218 are subject to *S*-glutathionylation by GSSG *in vitro*.

**FIGURE 5:**
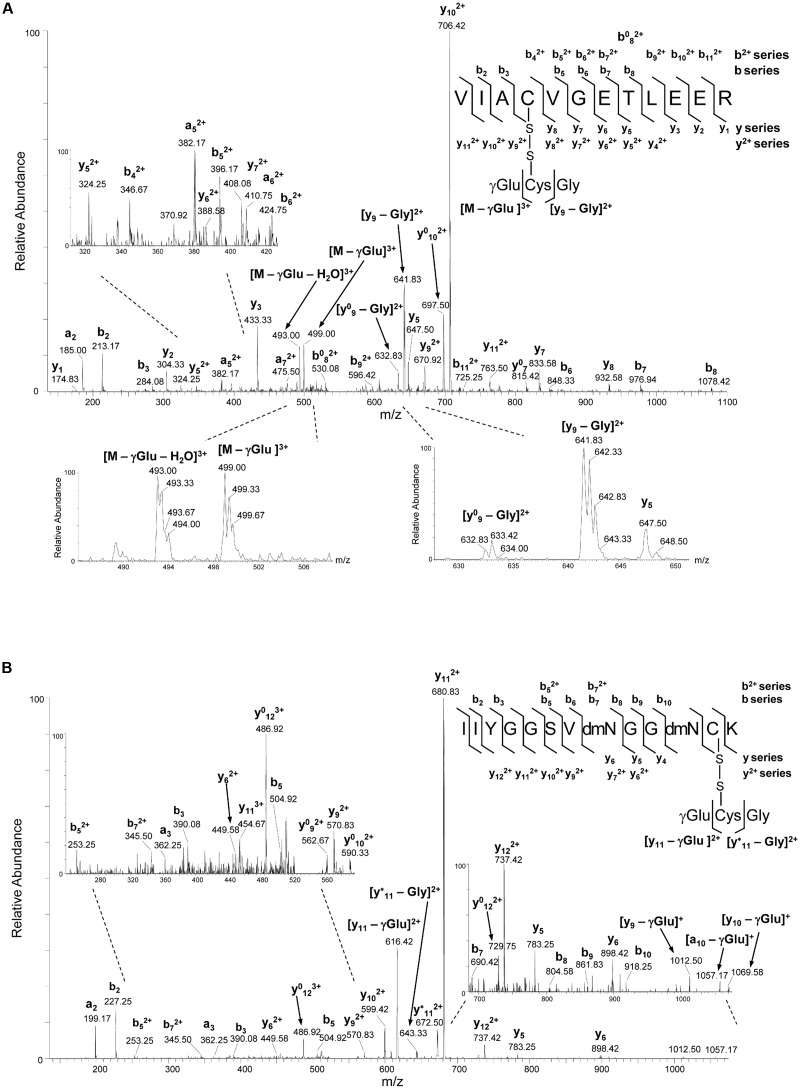
**NanoLC-MS/MS analysis of cTPI tryptic peptides modified by GSSG. (A)** CID MS/MS fragmentation spectrum of the precursor ion at *m/z* of 542.57^3+^ corresponding to *S*-glutathionylated peptide with the sequence ^124^VIACVGETLEER^135^. The detection of ion at *m/z* 499.00 indicate the loss of γGlu (-129 Da) from the triply charged precursor resulting in the ion [M – γGlu]^3+^, while the concomitant loss of water (-18 Da) resulted in the ion at m/z 493.00 corresponding to [M – γGlu – H_2_O]^3+^. Sequence specific y- and b-type fragment ion signals of different charge states (+1 and +2) identifying the peptide, and metastable decomposition product ions resulting from further internal fragmentation of glutathione adduct by elimination of γGlu or Gly residues are indicated. The peaks denoted y^0^ and b^0^ are the result of water (-18 Da) loss from corresponding ion. Spectral portions containing signature ions resulting from the loss of γGlu or Gly residues in glutathione with partly isotopically resolved peaks indicating ions charge state are shown. **(B)** CID MS/MS fragmentation spectrum of the precursor ion at *m/z* of 530.21^3+^ corresponding to *S*-glutathionylated peptide with the sequence ^207^IIYGGSVNGGNCK^219^. The expanded regions of the product ion spectra showing detailed fragmentation pathways of *S*-glutathionylated peptides are given on the left side of panel **(A)** and on both left and right sides of panel **(B)**. Product maps indicating the cleavage sites in CID MS/MS are given on the right side of panels **(A,B)**.

The CID MS/MS fragmentation spectra of precursor ions corresponding to *S*-glutathionylated forms of peptides with sequence ^124^VIACVGETLEER^135^ (**Figure 5A**) and ^207^IIYGGSVNGGNCK^219^ (**Figure 5B**) provided further specific information for the amino acid composition and identity of the two *S*-glutathionylated peptides in cTPI as well as for the site-specific assignment of this modification. The signature neutral loss of 129 Da was observed from the precursor [M – γGlu]^3+^ ion at *m/z* 542.17^3+^ characteristic to the loss of γ-glutamyl moiety from glutathione adduct (**Figure 5A**). A number of additional high intensity internal fragment ions were present in the product ion spectra of both modified peptides. They were the results of glutathione components γGlu- and Gly-residue loss from y-type ions upon collision in the mass spectrometer (**Figures 5A,B**). These ions provided further structural information about the nature of the adduct, thus, confirming the presence of glutathione in these peptides.

We performed cTPI sequence comparisons between different photosynthetic species using Clustal W (Larkin et al., 2007) (Supplemental Figure S6). Four Cys residues were found to be strictly or semi-conserved in cTPI sequences. *A. thaliana* Cys13 and Cys127 were strictly conserved while Cys67 and Cys218 were semi-conserved between different photosynthetic organisms. Interestingly, the Cys residue corresponding to Cys127 is present in the pTPI sequence of *A. thaliana*, but Cys218 is absent from the chloroplastic enzyme.

### Analysis of Cys127 and Cys218 by Site-Directed Mutagenesis

Site directed mutagenesis was performed to obtain three recombinant cTPI mutants (C127S, C218S, C127/218S) in which selected Cys residues were replaced by a Ser residue. Recombinant cTPI mutants were purified as described for the WT protein to obtain a homogeneous purified protein of each mutant (Supplemental Figure S7). Mutation of the targeted Cys residues altered cTPI activity distinctively. A loss of more than 97% of specific activity was observed for C127S and C127/218S mutants (**Table 1**). In the C218S mutant, loss of specific activity was less striking (around 21%). Interestingly, the *K*_m_ value for GAP was lower in the C127S mutant (**Table 1**).

**Table 1:** Kinetic analysis of recombinant WT and mutant cTPI proteins.

Protein	Specific activity (μmol min^-1^ mg^-1^)	*k*_cat_ (s^-1^)	*K*_m_ (mM)	*k*_cat_/*K*_m_ (×10^3^ M^-1^s^-1^)
WT	6580 ± 520	3406 ± 269	1.32 ± 0.06	2580 ± 321
C127S	136 ± 14	70.4 ± 7.4	0.48 ± 0.04	147 ± 28
C218S	5197 ± 140	2690 ± 73	1.68 ± 0.03	1601 ± 72
C127/218S	28.6 ± 1.6	14.8 ± 0.8	0.50 ± 0.06	29.6 ± 5.2

*Activities were measured using a coupled enzymatic assay in the presence of 5 mM DTT.*

**FIGURE 6:**
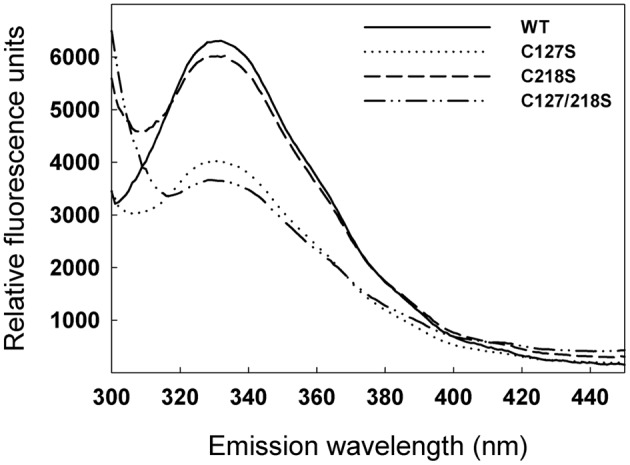
**Effect of C127S and C218S mutations on the fluorescence emission spectra of the cTPI recombinant protein.** Fluorescence emission spectra of recombinant WT and mutant cTPI proteins were recorded using an excitation wavelength of 274 nm.

In order to investigate a possible difference in protein conformation in the mutants compared to the WT, fluorescence emission spectra were studied. Recombinant proteins were excited at 274 nm and intrinsic fluorescence emission was recorded between 300 nm and 450 nm. The spectra of WT and the C218S mutant were relatively similar and differed from those of C127S and C127/218S, in particular with respect to the intensity of the peak between 320 and 340 nm (**Figure 6**).

**FIGURE 7:**
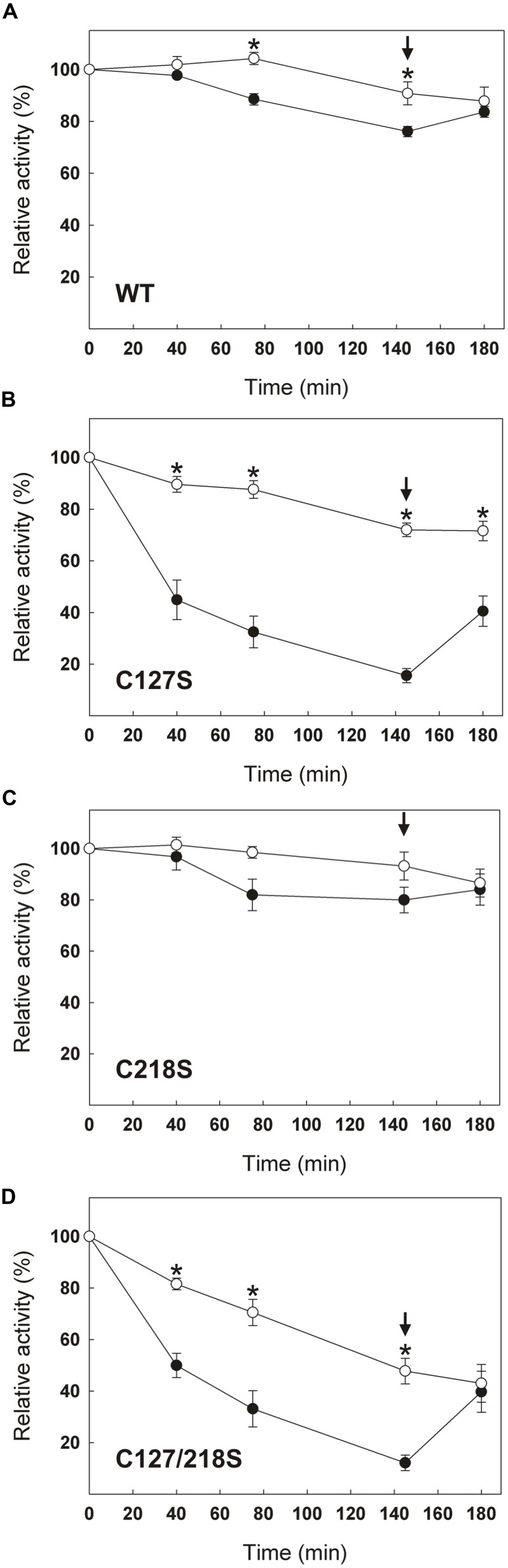
**Effect of C127S and C218S mutations on the stability of cTPI activity in presence or absence of DTT.** Recombinant **(A)** WT, **(B)** C127S, **(C)** C218S, or **(D)** C127/218S cTPI enzymes were incubated with (*white circles*) or without (*black circles*) 5 mM DTT. Activity was measured at different time points. A star indicates a significant difference between treatments (Student’s *t*-test, *P* < 0.05). After 145 min (*black arrow*), the samples incubated in absence of reductant received DTT (10 mM final concentration) and TPI activity was measured at 180 min. Data points are mean ± SE (*n* ≥ 3).

In addition to changes in their specific activity and fluorescence spectrum, C127S and C127/218S mutants were much less redox stable over time than the WT and the C218S mutant. Monitoring the time-dependent evolution of cTPI activity in the presence or absence of 5 mM DTT showed that the WT (**Figure 7A**) and the C218S (**Figure 7C**) mutant were more stable when incubated with DTT indicating that cTPI was sensitive to spontaneous oxidation. The two proteins kept more than 75% of their initial activity after 145 min when incubated without DTT. In contrast, within the same time frame, the C127S (**Figure 7B**) and C127/218S (**Figure 7D**) mutants lost 80% of their initial activity in absence of DTT. The activity of C127S and C127/218S mutants did not recover completely even after the addition of DTT. Due to the presence of DTT in these samples, the decrease in activity is probably not related to a spontaneous oxidation and could perhaps be due to an intrinsically less stable protein structure.

### Effect of Glutathione on cTPI Mutants

**FIGURE 8:**
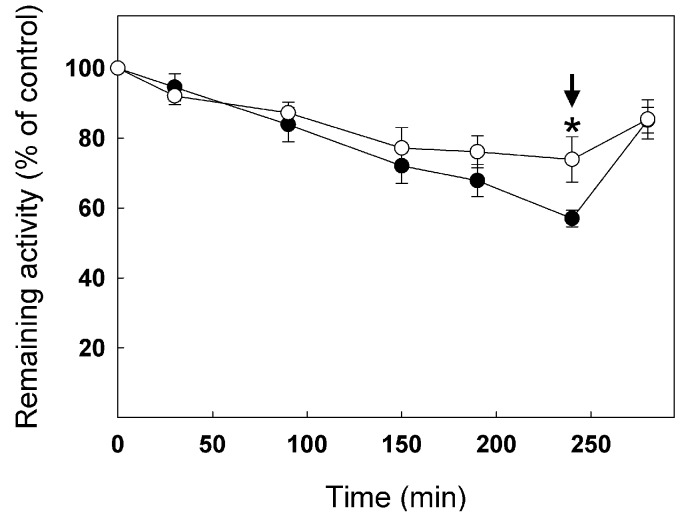
**Inhibition of TPI activity by GSSG in WT and C218S mutant.** Recombinant WT (*black circles*) and C218S mutant (*white circle*) cTPI proteins were incubated with 100 μM GSSG. TPI activity was measured at different incubation times. A star indicates a significant difference between treatments (Student’s *t*-test, *P* < 0.05). After 240 min (*black arrow*), both samples received 10 mM DTT before TPI activity was measured. Activity (mean ± SE, *n* = 3) is expressed as percentage of the control (untreated) sample remaining activity in order to take into account the loss of cTPI activity upon dilution without reductant.

Because the C127S mutation leads to very low stability of enzyme activity and high inhibition due to spontaneous oxidation over time, it was not possible to test the effect of GSSG on the activity of the C127S and C127/218S mutants. **Figure 8** shows inhibition of WT cTPI and C218S mutant by 100 μM GSSG over time. The C218S cTPI mutant behaved similarly to the WT, except that it significantly retained more activity than the WT after 240 min of incubation. Activity was partially restored by 10 mM DTT for both WT and C218S samples. These results show that C218S is sensitive to inhibition by GSSG. Thus, in *A. thaliana*, we observed that mutation of Cys218 does not confer resistance of cTPI activity to inhibition by GSSG.

**FIGURE 9:**
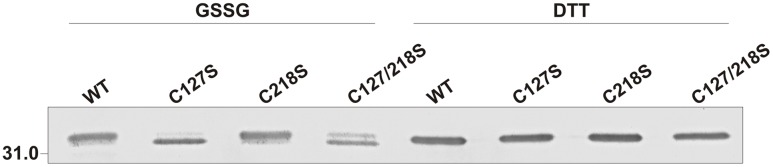
**Effect of GSSG on recombinant WT and mutant cTPI proteins mobility in non-reducing SDS-PAGE.** Enzymes were incubated with 1 mM GSSG or 15 mM DTT. The figure shows immunoblot analysis after non-reducing SDS-PAGE of recombinant cTPI (50 ng). The identity of the protein is indicated on top of each lane.

We performed non-reducing SDS-PAGE followed by anti-cTPI Western blot with the different cTPI mutants to visualize the effect of GSSG on the oxidation state of cTPI (**Figure 9**). Upon incubation with GSSG, two bands were observed indicating differences in electrophoretic mobility. Addition of DTT to the samples led to resolution of the proteins as a single band. The band with higher mobility suggests the formation of intramolecular disulfide bond in cTPI structure. After GSSG treatment, the WT protein and the C218S mutant showed a barely visible band with higher mobility, while most of the signal was associated with the upper band. On the contrary, the C127S and C127/218S mutants displayed a much higher signal corresponding to the high mobility band when incubated with GSSG. In this case, it remains unclear whether GSSG promotes formation of intramolecular disulfide bond in cTPI structure and if it is related to *S*-glutathionylation.

**FIGURE 10:**
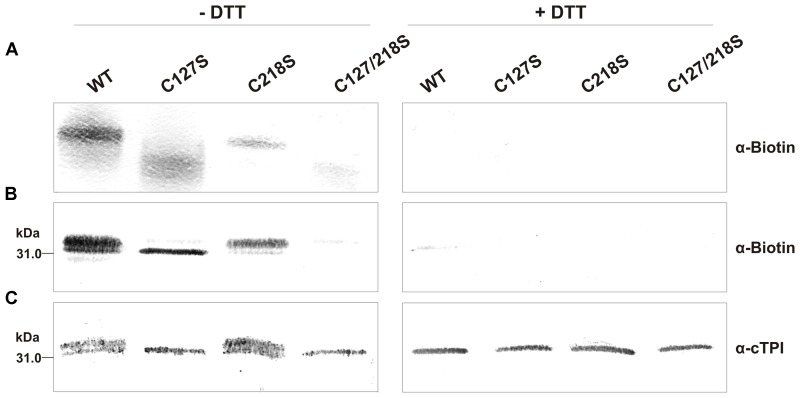
**BioGEE/Streptavidin detection and immunoblot analysis of recombinant WT and mutant cTPI proteins after native and SDS-PAGE.** BioGEE detection by streptavidin conjugated with alkaline phosphatase after **(A)** Native PAGE analysis of recombinant proteins (300 ng) and Western blotting or **(B)** SDS-PAGE analysis of recombinant proteins (40 ng) and Western blotting. **(C)** Immunoblot analysis after SDS-PAGE of recombinant proteins (80 ng) using an isoform-specific cTPI antibody. Enzymes were incubated in absence (left panels) or presence (right panels) of 50 mM DTT in a buffer containing: **(A)** 100 mM Tris-Cl pH 7.8, 0.5 mM BioGEE, 0.1 mM H_2_O_2_ and (for **B,C** only) 0.005% (w/v) BSA.

We next incubated the WT and mutant proteins with 0.5 mM of BioGEE and 0.1 mM of H_2_O_2_ followed by Western blot analyses using streptavidin detection (**Figures 10A,B**) and anti-cTPI antibody (**Figure 10C**). These experiments were performed with or without DTT to observe the reversibility of the modification. Streptavidin detection showed the presence of bands with different intensity for WT, C127S and C218S, but no significant signal for the C127/218S mutant. Most of the BioGEE signal was removed following addition of DTT. These results suggest that Cys127 and Cys218 are the major targets of *S*-glutathionylation in cTPI. The anti-cTPI detection (**Figure 10C**) showed a pattern similar to that previously observed in **Figure 9**, indicating differences in cTPI mobility due to the treatment. In these conditions, WT and C218S form two bands while only the bottom band is visible for C127S and C127/218S.

## Discussion

*S*-Glutathionylation has recently emerged as a PTM that takes part in redox signaling in photosynthetic organisms. Enzymes from carbon metabolism constitute major targets for redox PTMs including *S*-glutathionylation. In the plant cytosolic compartment, many glycolytic enzymes have been reported to be inhibited by this modification. Among these targets, enzymatic activities of cTPI, aldolase and GAPDH (GAPC1 and GAPC2) were reported to be inhibited by *S*-glutathionylation (Ito et al., 2003; Holtgrefe et al., 2008; van der Linde et al., 2011; Bedhomme et al., 2012). Low cTPI activity in transgenic *Solanum tuberosum* roots carrying cTPI antisense constructs has been associated with increased carbon flux through the PPP (Dorion et al., 2012; Valancin et al., 2013). The effect of TPI inhibition in cell response to oxidant treatment has also been well documented in non-plant systems. Reduced TPI activity has been linked to resistance against diamide, rerouting of metabolic flux through PPP and accumulation of PPP metabolites in *Saccharomyces cerevisiae* and *C. elegans* (Ralser et al., 2007; Grüning et al., 2014). Interestingly, reduction of GAPDH activity, a well-known redox regulated enzyme, led to similar observations (Ralser et al., 2007).

### Recombinant cTPI Is Sensitive to Oxidant and *S*-Glutathionylation Treatments

In this work, we incubated cTPI with NEM, H_2_O_2_ and diamide with or without addition of GSH to study the effect of derivation of Cys residues on cTPI activity (**Figure 2**). Diamide is a chemical thiol-specific oxidant whereas H_2_O_2_ is a physiological oxidant. NEM is an alkylating reagent that blocks thiol groups irreversibly. Here, we showed that alkylation of cTPI Cys residues by NEM led to inhibition of the enzyme. Alkylation can be buffered by GSH when its concentration is higher than NEM thus protecting cTPI from inactivation. In contrast, we observed that addition of GSH did not protect cTPI from oxidation by H_2_O_2_. This suggests the presence of nucleophilic Cys residues in cTPI sequence. It is known that H_2_O_2_ oxidizes preferentially nucleophilic thiol groups. GSH has a high p*K*_a_ in solution and does not constitute a major target for spontaneous oxidation by ROS (Zaffagnini et al., 2012d). However, the p*K*_a_ of protein Cys residues can be lowered by their microenvironment thus stabilizing the thiolate anion which is more prone to react with ROS (Zaffagnini et al., 2012d). Advanced oxidized forms of Cys127 (sulfinic and sulfonic acid) were detected by mass spectrometry (Supplemental Figure S5) showing high reactivity of this residue.

Diamide is a thiol-specific oxidant that has been used in mammalian (Fratelli et al., 2002; Lock et al., 2011) and photosynthetic (Michelet et al., 2008) cells to promote *S*-glutathionylation *in vivo.* Diamide is known to react rapidly with cellular GSH to form GSSG (Kosower and Kosower, 1995). In rat erythrocytes, diamide was shown to induce *S*-glutathionylation by first reacting with hemoglobin and then promoting *S*-glutathionylation with GSH (Di Simplicio et al., 1998; Ghezzi and Di Simplicio, 2007). Coupled with GSH, diamide has also been used *in vitro* to show the inhibition of a mammalian protein tyrosine phosphatase by *S*-glutathionylation (Barrett et al., 1999). Here, we show that incubation of cTPI with GSH + diamide lead to rapid and almost total inhibition of cTPI activity while diamide alone as limited effects on cTPI activity especially in a short period of time (**Figure 2**). We suggest that, in the conditions used in our experiments, diamide could react rapidly with GSH to form a reactive diamide-GS conjugate as described in Kosower and Kosower (1995). This intermediate could then spontaneously react with reduced thiols present in the samples thus inducing protein *S*-glutathionylation and inhibition of cTPI activity. Being already oxidized, GSSG cannot form a reactive diamide-GS conjugate which can explain why incubation of cTPI with diamide + GSSG does not affect activity within 5 min. Although it does not seem to induce diamide-mediated *S*-glutathionylation, GSSG is known to induce *S*-glutathionylation by thiol disulfide exchange (Ghezzi, 2005). However, in our experiments inhibition of cTPI activity with GSSG was slower than that observed by treatment with diamide + GSH.

Activity of recombinant *A. thaliana* cTPI was previously reported to be inhibited by 2.5 mM GSSG (Ito et al., 2003). However, this inhibition has never been studied under more physiological concentrations of GSSG. GSSG concentrations in plant cells cytosol are very low under basal conditions. *In situ* experiments using a ratiometric redox-sensitive green fluorescent protein suggested that cytosolic GSSG concentrations in normal conditions are found in the low nanomolar range in *A. thaliana* (Meyer et al., 2007). Such reducing conditions could be explained by the reduction of GSSG by glutathione reductase activity but also by the sequestration of GSSG, particularly in the vacuole (Meyer et al., 2007; Noctor et al., 2013). By using a GSSG-accumulating catalase-deficient (*cat2*) *A. thaliana* mutant as a model for oxidative stressed cells, it was estimated that GSSG concentrations could reach the micromolar range in stressed plant cytosol by assuming that 99.9% of the GSSG is sequestrated inside the vacuole (Noctor et al., 2013). These data suggest that cytosolic GSSG concentrations remain low in plant cells even under oxidative stress. In this work, we showed that cTPI activity can be affected by micromolar concentrations of GSSG *in vitro* (**Figure 3**). These data support the view that cTPI activity could be regulated by conditions similar to those prevailing under oxidative stress. As previously mentioned, treatment of proteins with GSSG can lead to *S*-glutathionylation by thiol-disulfide exchange (Ghezzi, 2005), but GSSG has also been reported to promote formation of other types of disulfide bonds (Cumming et al., 2004). Moreover, catalysis of thiol disulfide exchange could also accelerate cTPI *S*-glutathionylation and inhibition. Indeed, a recent study has shown that *S*-glutathionylation of BAK1 by GSSG can be catalyzed by GRXC2 in *A. thaliana* (Bender et al., 2015). The authors showed that lower concentrations of GSSG were required for inhibition of the BAK1 kinase activity in the presence of GRXC2. However, catalysis of *S*-glutathionylation is still poorly understood in plants and needs further investigation.

### *S*-Glutathionylation of Recombinant cTPI Is Reversed by GRXs

Since deglutathionylation is known to be catalyzed by GRXs (Rouhier et al., 2008; Bedhomme et al., 2012) we expressed and purified recombinant GRXC1 and GRXC2 from *A. thaliana* (Supplemental Figure S4). Determination of *k*_cat_ values showed that they were similar to those obtained for poplar homologs *Pt*GRXC1 and *Pt*GRXC2 (Couturier et al., 2013). Our results were also in the range of activities measured for *C. reinhardtii* GRX1 and GRX2, two class I GRXs predicted to be cytosolic (Zaffagnini et al., 2008). However, *k*_cat_ value reported here for *A. thaliana* GRXC1 (*k*_cat_ = 8.0 ± 0.9 s^-1^) was lower than that reported by Riondet et al. (2012) (38.91 ± 0.43 s^-1^). Both *A. thaliana* GRXC1 and GRXC2 are class I GRXs (CPYC-like motif) well known for their reductase activity and localized in the cytosol (Riondet et al., 2012; Couturier et al., 2013). Despite the well-known ability of GRXC1 but not GRXC2 to bind [2Fe-2S] clusters (Rouhier et al., 2007, 2010; Riondet et al., 2012; Couturier et al., 2015), both GRXC1 and GRXC2 are suggested to have redundant functions in plant cells (Riondet et al., 2012). In our experiments, both GRXC1 and GRXC2 were able to restore cTPI activity with a similar efficiency. Both GRXs were dependent on GRS for deglutathionylation of cTPI (**Figure 4**). Similar reactivation experiments have previously shown GRXs-mediated deglutathionylation. Cytosolic enzyme GAPC1 from *A. thaliana* was shown to be inactivated by *S*-glutathionylation and rescued by cytosolic poplar *Pt*GRXC1 through a monothiol mechanism (Bedhomme et al., 2012). Other experiments showed reactivation of *S*-glutathionylated isocitrate lyase from *C. reinhardtii* by *Cr*GRX1, *Cr*GRX2 (Gao et al., 2010) and poplar chloroplastic class I GRXS12 (Zaffagnini et al., 2012c). Overall, our results suggest that cTPI activity can be inhibited by *S*-glutathionylation and rescued by cytosolic GRXs such as GRXC1 and GRXC2.

### cTPI Cys127 and Cys218 Residues Are Targets of Glutathione and Other Redox PTMs

In animals, the Cys residue from *Gallus gallus* TPI corresponding to Cys127 was described to be sensitive to oxidation leading to inhibition of the enzyme (Tang et al., 1990). Subsequently, a proteomic survey of human T lymphocytes identified TPI as a target of *S*-glutathionylation, but the modified Cys were not identified (Fratelli et al., 2002). Recently, the Cys127 residue of photosynthetic cyanobacterium *Synechocystis* sp. PCC6803 TPI was identified as a target of *S*-glutathionylation (Chardonnet et al., 2014). In higher plants, cTPI is known to be *S*-glutathionylationed (Ito et al., 2003), however, the modified amino acids were not identified. Here we used nanoLC-MS/MS analysis to show that recombinant cTPI from *A. thaliana* was modified by GSSG on residues Cys127 and Cys218 (**Figure 5**). Interestingly, Cys218 is located next to a Lys residue (Lys219) (Supplemental Figure S5) which could contribute to the stabilization of the thiolate anion. Plant cTPI was also found to be modified by *S*-nitrosylation (Romero-Puertas et al., 2008; Fares et al., 2011). Moreover, Cys127 and Cys218 residues were shown to be *S*-nitrosylated in *A. thaliana* cell culture (Fares et al., 2011). Cys residues *S*-nitrosylation is a spontaneous redox PTM induced by reactive nitrogen species that shows some similarities with sulfenic acid formation. *S*-nitrosylated Cys thiols are considered to be reactive and can thus be modified by GSH leading to *S*-glutathionylation (Martínez-Ruiz and Lamas, 2007). Considering our results, plant cTPI Cys127 and Cys218 residues appear to be major targets of redox modification including *S*-glutathionylation.

### Mutation Cys127 and Cys218 Affects cTPI Activity, Structure, Stability, and Interaction with Glutathione

*Arabidopsis thaliana* cTPI contains four Cys residues (Supplemental Figure S5), none of which is known to be directly involved in the TPI catalytic site (Komives et al., 1991; Zaffagnini et al., 2014). In the structure of *C. reinhardtii* pTPI and in *G. gallus* TPI, Cys residues corresponding to Cys127 are located near the catalytic site, while Cys residues corresponding to Cys218 are exposed to the solvent (Banner et al., 1975; Zaffagnini et al., 2014). Being close to the active site, modification of Cys127 is more likely to affect cTPI activity. Our data demonstrate that mutation of Cys127 to a Ser residue lead to strong perturbation of *A. thaliana* cTPI kinetics parameters (**Table 1**). Nonetheless, mutation of Cys126 (corresponding to Cys127) to a Ser residue in *S. cerevisiae* TPI was reported to have a lower impact on enzyme kinetics (González-Mondragón et al., 2004). This mutation caused a four-fold decrease in yeast TPI *k*_cat_ while we observed a 48-fold decrease for the same mutation in *A. thaliana* cTPI. Yeast TPI C126S mutant was also reported to be less stable and more subject to irreversible denaturation than the WT. Similarly, mutation of Cys126 to a Ser in TPI from *P. falciparum* caused a five-fold decrease of activity. It was also proposed that mutation of this Cys residue decreased the dimer stability at elevated temperatures (Samanta et al., 2011). Later, it was suggested that the low stability of yeast C126S was due to penetration of water molecules in the region near the residue (Hernández-Santoyo et al., 2012). It should be noted that the amino acids sequence of *S. cerevisiae* TPI has no Cys corresponding to Cys218 in *A. thaliana* cTPI.

While it was impossible to test the effect of GSSG on C127S and C127/218S mutant due to their low stability, we showed that incubation of C218S with GSSG mutant caused a decrease in activity similar to that observed for the WT (**Figure 8**). These results differ from data obtained with *Giardia lamblia* TPI. In this latter case, inactivation was mostly caused by derivatization of Cys222 (corresponding to Cys218 in *A. thaliana* cTPI). Indeed, the activity of the C222A mutant was completely resistant to inactivation by sulfhydryl reagents such as 2-carboxyethyl methanethiosulfonate and 5,50-dithio-bis (2-nitrobenzoic acid) while the WT and all other mutants for Cys residues were strongly inhibited by these reagents (Enríquez-Flores et al., 2011).

Difference in electrophoretic mobility of oxidized and reduced TPI has already been observed in chicken where the oxidized form was suggested to have lower catalytic activity (Tang et al., 1990). Here, we found that exposure of cTPI to GSSG or H_2_O_2_ and BioGEE led to the formation of two bands with different mobility only in the absence of DTT (**Figures 9** and **10**). Surprisingly, mutation of both targets of *S*-glutathionylation, Cys127 and Cys218, did not confer oxidant resistance. On the contrary, C127S and C127/218S mutants were less stable (**Figure 7**) and more likely to form a higher mobility band corresponding to an oxidized form of cTPI. However, the C127/218S mutant does not seem to be significantly modified by BioGEE since both *S*-glutathionylation sites are mutated. It still remains unclear whether Cys13 and Cys67 residues are redox sensitive in cTPI. Formation of a higher mobility band for the oxidized C127/218S mutant and spontaneous loss of activity could be attributed to the formation of an intramolecular disulfide bond between the two remaining Cys residues. Moreover, rapid formation of disulfide bond between Cys13 and Cys67 could also prevent interaction of the C127/218S mutant with BioGEE as observed here. This redox sensitivity could be linked to perturbations in cTPI structure due to the mutation of the strictly conserved Cys127 residue. Differences in fluorescence emission spectra provide an indication of alterations in protein structure. A mutation of the redox sensitive Cys127 seemed to significantly affect protein fluorescence and kinetics parameters. Given our results, we hypothesize that the C127S substitution had more impact on protein structure than the C218S mutation. This is in agreement with results of specific activity showing a drastic decrease in cTPI activity in the C127S mutant protein.

## Conclusion

In the present study, we showed that recombinant cTPI from *A. thaliana* is sensitive to inhibition by different treatments promoting Cys oxidation or *S*-glutathionylation. Inhibition of cTPI by *S*-glutathionylation was reversed by the activity of recombinant GRXC1 and GRXC2 from *A. thaliana* in the presence of a GRS. We used nanoLC-MS/MS to identify Cys127 and Cys218 as the two residues targeted by GSSG. Mutation of targeted Cys appeared to affect cTPI structure, activity and redox stability. When incubated with BioGEE, the cTPI mutant that lacks both targeted Cys residues did not show a significant signal on nitrocellulose membrane after streptavidin detection.

We propose that *S*-glutathionylation of cTPI in *A. thaliana* could be involved in the response to oxidizing conditions in the cytoplasmic compartment. In this hypothesis, a stress-induced *S*-glutathionylation would lead to a decrease in the activity of cTPI and other glycolytic enzymes such as cytosolic GAPDH and aldolase. This mechanism could lead to an increase in carbon flux through the PPP and enhanced production of NADPH used for restoration of GSH.

## Author Contributions

SDu, NB, GP, and SDo performed the experimental work; SDu, NB, and JR planned the experiments, interpreted the data and wrote the paper; all the authors discussed the data and revised the manuscript prior to submission.

## Conflict of Interest Statement

The authors declare that the research was conducted in the absence of any commercial or financial relationships that could be construed as a potential conflict of interest.

## Abbreviations

ABRC: *Arabidopsis* Biological Resource Center
BAK1: Brassinosteroid insensitive 1-Associated receptor-like Kinase 1
BioGEE: biotinylated glutathione ethyl ester
BioGSSG: biotinylated oxidized glutathione
BSA: bovine serum albumin
CID: collision-induced dissociation
cTPI: cytosolic triosephosphate isomerase
DHAP: dihydroxyacetone phosphate
GAP: glyceraldehyde-3-phosphate
GAPDH: GAP dehydrogenase
GRXC1: glutaredoxin C1
GRXC2: glutaredoxin C2
GRS: GSH-regeneration system
GSH: reduced glutathione
GSNO: *S*-nitrosoglutathione
GSSG: oxidized glutathione
HED: 2-hydroxyethyl disulfide
H_2_O_2_: hydrogen peroxide
LC-MS/MS: liquid chromatography-tandem mass spectrometry
NEM: *N*-ethylmaleimide
PPP: pentose phosphate pathway
PTM: post-translational modification
pTPI: plastidic TPI
ROS: reactive oxygen species
TRX: thioredoxin
WT: wild-type
