# Supplementary material for: Cytosolic Triosephosphate Isomerase from Arabidopsis thaliana Is Reversibly Modified by Glutathione on Cysteines 127 and 218
Source: Front Plant Sci. 2016 Dec 22;7:1942. doi: 10.3389/fpls.2016.01942 (PMC5177656; doi:10.3389/fpls.2016.01942)
Supplement: Supplementary file 1 [file Presentation_1.PDF]

Supplementary Material

Cytosolic triosephosphate isomerase from *Arabidopsis thaliana* is reversibly modified by glutathione on Cysteines 127 and 218

Sébastien Dumont, Natalia V. Bykova, Guillaume Pelletier, Sonia Dorion and Jean Rivoal*

***Correspondence :**
Jean Rivoal
jean.rivoal@umontreal.ca

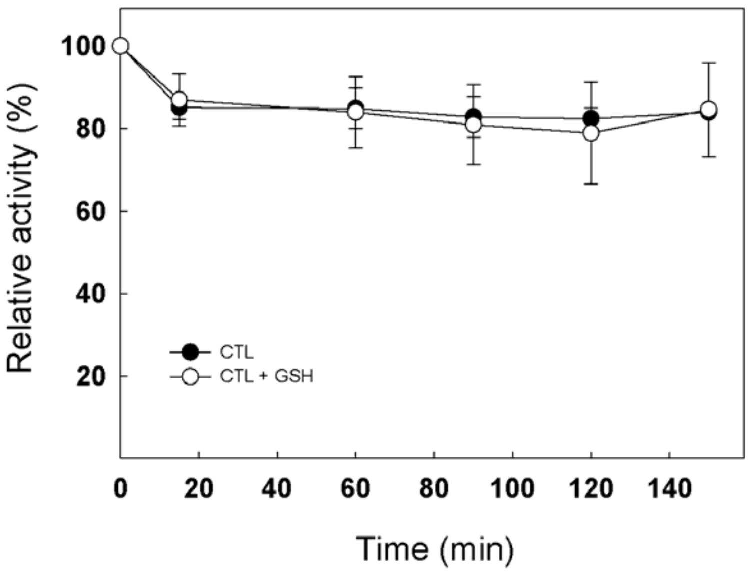

Supplemental Figure S1. Instability of stored cTPI upon dilution. Purified recombinant cTPI stored in 50% (v/v) glycerol was diluted to 0.2 µg/ml in 100 mM Tris-Cl pH 7.8, 0.005% (w/v) BSA with or without addition of 1 mM GSH. The enzyme was incubated at room temperature. This graph represents relative activity of cTPI control samples for the time points used in **Figure 2A and 2B**.

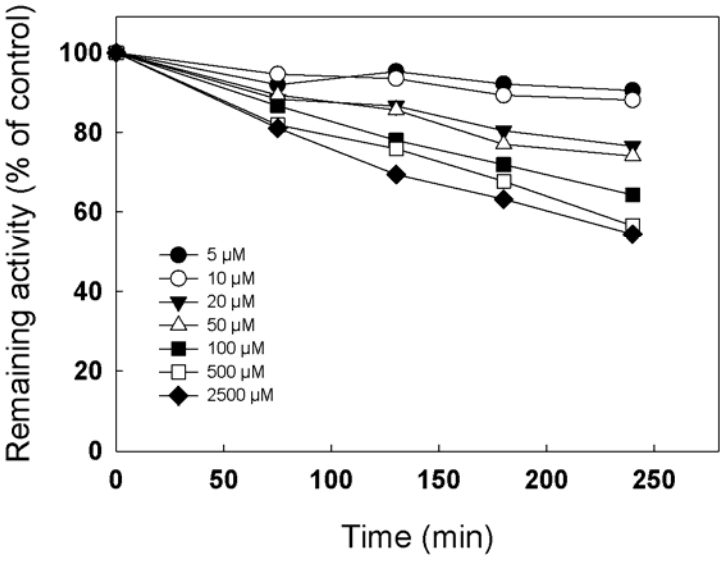

Supplemental Figure S2. Inhibition of cTPI activity by different concentrations of GSSG in function of the time. Recombinant cTPI was incubated with different GSSG concentrations. TPI activity is plotted as a function of incubation time (with the same data presented in **Figure 3**). Activity is expressed as percentage of the control (untreated) sample remaining activity in order to take into account the loss of cTPI activity upon dilution without reductant.

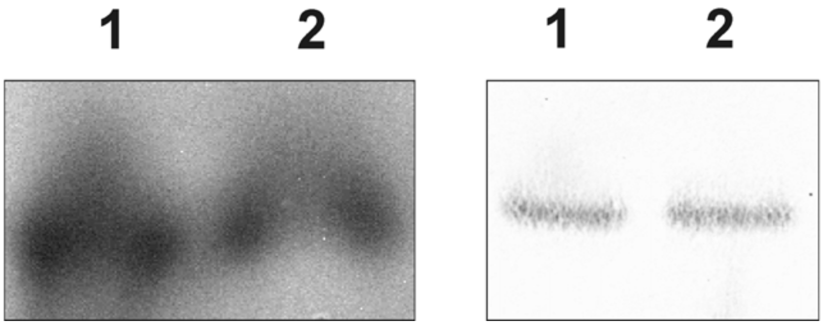

Supplemental Figure S3. cTPI activity staining after native PAGE. Native PAGE of 50 ng cTPI followed by activity staining (left panel) and immunoblot using an isoform specific cTPI antibody (right panel). cTPI was incubated for 180 min without GSSG (lane 1) or with 2.5 mM GSSG (lane 2) prior to electrophoretic separation.

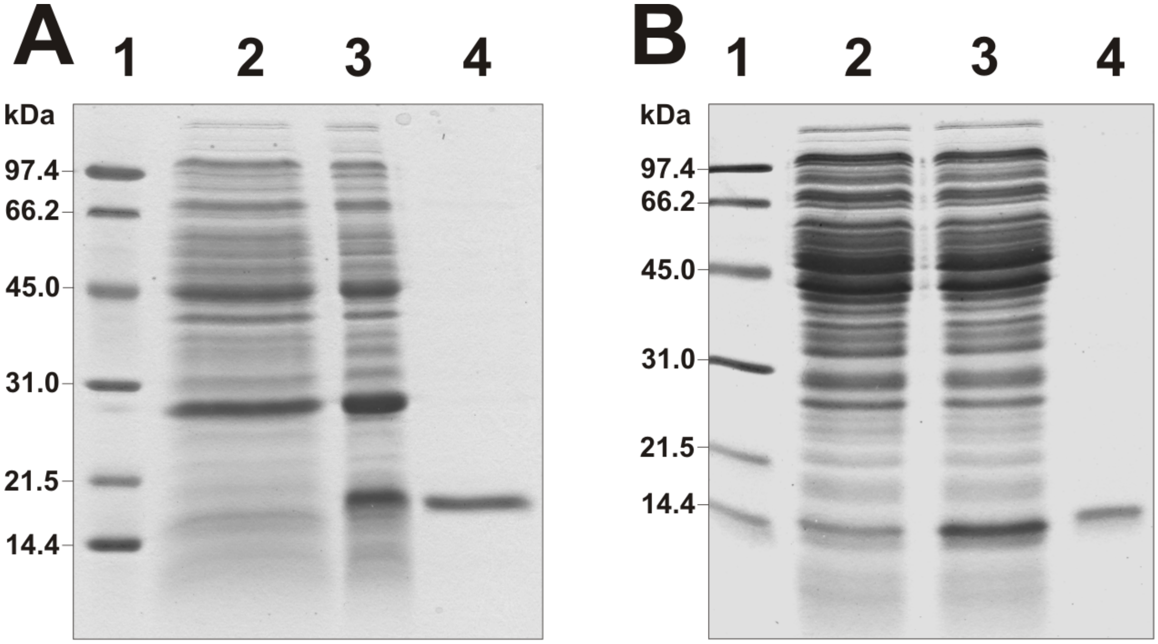

Supplemental Figure S4. Purification of His-tagged recombinant GRXC1 and GRXC2. SDS-PAGE analysis of purification of recombinant GRXs. Lane 1, molecular weight standards; lane 2, *E. coli* protein extract without induction; lane 3, *E. coli* protein extract after isopropyl ß-D-thiogalactoside induction; lane 4, affinity-purified recombinant **(A)** GRXC1 and **(B)** GRXC2.

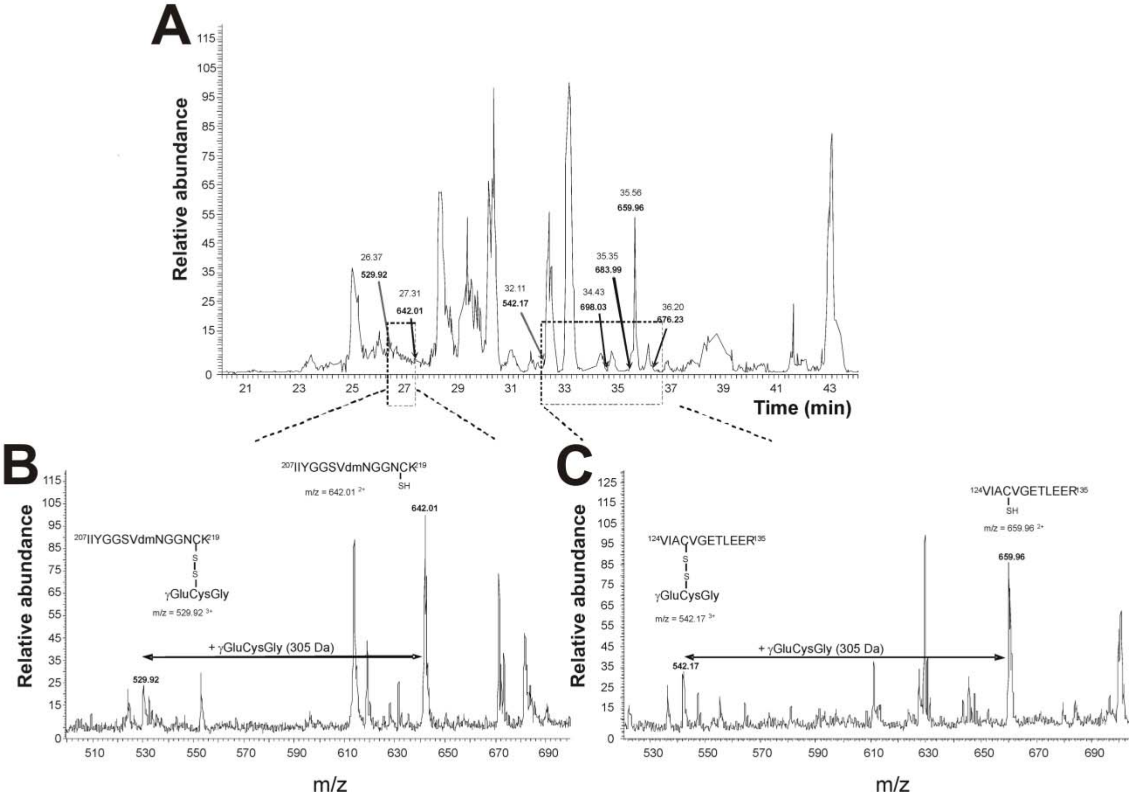

Supplemental Figure S5. LC-MS/MS analysis of cTPI tryptic peptides with and without prior incubation with GSSG. (A) The base peak chromatogram of MS survey scans during LC-MS/MS analysis of tryptic digest obtained from 36 pmol of cTPI. Arrows indicate retention times and *m/z* values of precursor ions corresponding to peptides with Cys oxidative modifications selected for data-dependent CID MS/MS analysis. The expanded regions of MS survey scans for retention time windows (dashed areas) with precursor ion peaks corresponding to unmodified (reduced thiol group Cys-SH) and *S*-glutathionylated peptides with the sequence ^{207}IIYGGSVNGGNCK^{219} **(B)** and sequence ^{124}VIACVGETLEER^{135} **(C)**. The delta mass differences of 305 Da between doubly charged and triply charged precursor ions resulting in the same MS/MS-derived peptide sequence corresponds to a unique signature of glutathione adduct. The peptide with sequence ^{124}VIACVGETLEER^{135} was also found in two other oxidized forms with Cys-SO_2H sulfinic acid (at *m/z* of 676.23^{2+}) and Cys-SO_3H sulfonic (at *m/z* of 683.99^{2+}).

```
A.thaliana        --markffvggnwkcngtaeevkkivntlneaqvpsqdvvevvvsppyvflplvkstlrs   58
S.tuberosum       --mgrtffvggnwkcngtseeikkivatlnagqvpsqdvvevvvsppfvflplvknelrs   58
Z.mays            --mgrkffvggnwkcngttdqvekivktlnegqvppsdvvevvvsppyvflpvvksqlrq   58
G.max             --mgrkffvggnwkcngtteevkkivttlneakvpgedvvevvvsppfvflpfvksllrp   58
P.vittata         --markffvggnwkcngtvaevnkivkllneadvpsedvvevvisppfvflpqvkavlrs   58
O.sativa          -maarkffvggnwkcngtgedvkkivtvlneaevpsedvvevvvsppfvflpqvkgllrp   59
C.variabilis      --markffvggnwkcngtvksneelvkvlnaaevpgtdvvevvvapaaghfpqvlsslrk   58
E.gracilis        --mprkffvggnwkcngtresiskiieefnkgpsvadadvevvigcpfvyadytreklrq   58
P.patens          magtgrffvggnwkcngtveslkklvdelnsakl--eedvdivvsppylyisqvlgsltk   58
                        ************  . .:::  :* .       *::*:.    .   .   *

A.thaliana        dffvaaqncwvkkggaftgevsaemlvnldipwvilghserrai--lnessefvgdkvay   116
S.tuberosum       dfhvaaqncwvkkggaftgevsadmlvnlgipwvilghserrai--lgesnefvgdkvay   116
Z.mays            efhvaaqncwvkkggaftgevsaemlvnlgvpwvilghserral--lgesnefvgdkvay   116
G.max             dfhvsaqncwvrkggaytgevsaemlvnlgipwviighserrql--lnelnefvgdkvay   116
P.vittata         dfavaaqnawvrkggaytgeisaemlinleipwvilghserral--lkesnefvadkvay   116
O.sativa          dfsvaaqncwvrkggaftgeisaemlvnlqvpwvilghserral--mgessdfvadkiay   117
C.variabilis      dfsvaaqncwvkkggaftgelsaemlkdlglpwvvlghserrhi--igesdefiadkvay   116
E.gracilis        dwalsvqncwigkggaftgeisaemikdagipwvilghserrhlpelkesdetvaikvay   118
P.patens          rievaaqncwtgkggaftgeisadqlvdggvkwviqghserrhv--igetdamigqksay   116
                     ::.**.*  ****:***:**: : :  : **: ****** :  : * .  :. * **

A.thaliana        alaqglkviacvgetleereagstmdvvaaqtkaiadrv--tnwsnvviayepvwaigtg   174
S.tuberosum       alsqglkviacvgetleqresgstmdvvaaqtkaiaerv--kdwsnvvvayepvwaigtg   174
Z.mays            alsqglkviacvgetleqreagstmdvvaaqtkaiaeki--kdwsnvvvayepvwaigtg   174
G.max             alqqglkviacigetleqreagtttavvaeqtkaiaaki--snwdnvvlayepvwaigtg   174
P.vittata         alsqglkviacigetleqreagetlnvvseqtkaiaeki--kdwgnvvlayepvwaigtg   174
O.sativa          alsqgikviacigetleqreagttmevvaaqtkaiaeki--sdwtnvvlayepvwaigtg   175
C.variabilis      algqglgviycigekleereagntmavnarqmqaladki--sdwskvvvayepvwaigtg   174
E.gracilis        alanglkvmacigelleereggqtqavnerqlkaiaaklkeedwknvviayepvwaigtg   178
P.patens          alsknlgviacvgekledreanrttdvvfeqlqayadav--sdwsnivvayepvwaigtg   174
                  ** : : *: *:** **:**.  *  *   * :* *  :   :* ::*:***********

A.thaliana        kvaspaqaqevhdelrkwlaknvsadvaattriiyggsvnggnckelggqadvdgflvgg   234
S.tuberosum       kvaspaqaqevhaelrkwlqanvsaevaastriiyggsvsganckelagqpdvdgflvgg   234
Z.mays            kvatpaqaqevhaslrdwlktnaspevaestriiyggsvtaanckelaaqpdvdgflvgg   234
G.max             kvatpaqaqevhadlrkwvhdnvsaevaasvriiyggsvnggnckelaaqpdvdgflvgg   234
P.vittata         kvatpvqaqevhadlrswlatnvssdvaesvriiyggsvnagncvelagqpdvdgflvgg   234
O.sativa          kvatpaqaqevhdglrkwlvtnvspavaestriiyrgsvnganckelaakpdvdgflvgg   235
C.variabilis      kvaspaqaqevhdelrkwlsanvspevaeatriiyggsvtaancgelagcpdidgflvgg   234
E.gracilis        kvatpeqaqevheqvrawvasnvspsvaaevrilyggsvtaknsaelagkpdvdgflvgg   238
P.patens          kvaspqqaqevhaairqwlkekispevssktriiyggsvngansaelatqedidgflvgg   234
                  ***:* ******  :* *:  : *  *:  .**:* ***.. *. **.   *:*******

A.thaliana        aslkp-efidiikaaevkksa--   254
S.tuberosum       aslkp-efidiikaaevkksa--   254
Z.mays            aslkp-efidiinaatvksa---   253
G.max             aslka-efvdiinaatvkkn---   253
P.vittata         aslka-efvdiirsalvsks---   253
O.sativa          aslkp-efvdiiksatvkssa--   255
C.variabilis      aslkp-efvdiikaaeksk----   252
E.gracilis        aslkp-efldivaaykhk-----   255
P.patens          aalkglefaticnavtakkavaa   257
                  *:**  **  *  :   .
```

Supplemental Figure S6. Alignment of cTPI sequences from different photosynthetic organisms. cTPI sequences have been obtained from NCBI resource centre. The sequences were aligned with Clustal W. Cys residues are highlighted. Sequence accession numbers are: *Arabidopsis thaliana*, NP_191104.1; *Solanum tuberosum*, NP_001305511.1; *Zea mays*, NP_001140424.1; *Glycine max*,

NP_001237472.1; *Pteris vittata* ADP21078.1, *Oryza sativa*, ADM86861.1; *Chlorella variabilis*, XP_005845877.1; *Euglena gracilis*, AAR04016.1; *Physcomitrella patens*, XP_001768780.1.

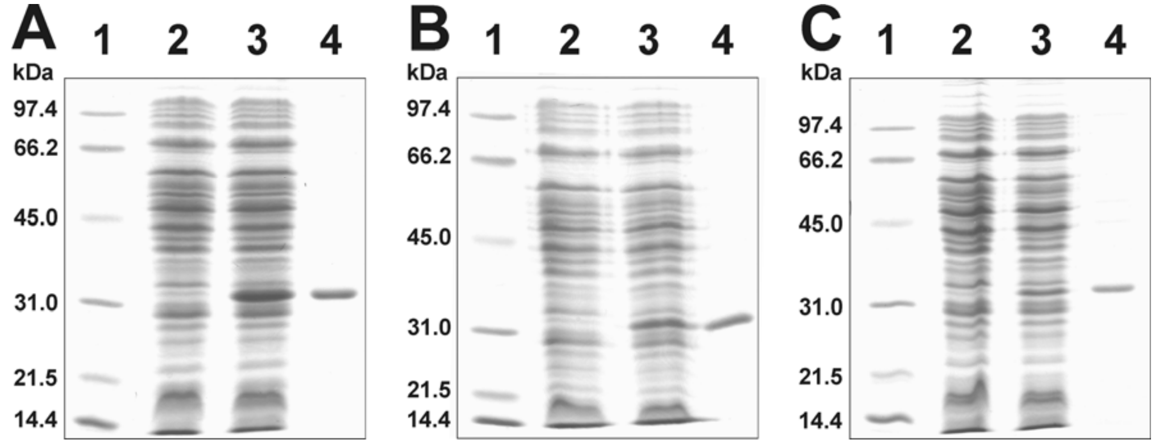

Supplemental Figure S7. SDS-PAGE analysis of the purification of His-tagged recombinant cTPI mutants. Purification steps of **(A)** C218S, **(B)** C127S, **(C)** C127/218S cTPI mutants on SDS-PAGE. Lane 1, molecular weight standards; lane 2, *E. coli* protein extract without induction; lane 3, *E. coli* protein extract after isopropyl ß-D-thiogalactoside induction; lane 4, affinity-purified recombinant cTPI.
